# Complete chloroplast genome sequence of *Heteroplexis sericophylla* (Asteraceae), a rare and vulnerable species endemic to China

**DOI:** 10.1080/23802359.2019.1680323

**Published:** 2019-10-23

**Authors:** Yancai Shi, Ying Zhang, Bingbing Liu

**Affiliations:** a Institute of Loess Plateau, Shanxi University, Taiyuan, Shanxi, China;; b Guangxi Institute of Botany, Guangxi Zhuang Autonomous Region and Chinese Academy of Sciences, Guilin, China

**Keywords:** *Heteroplexis*, chloroplast genome, phylogenetic analysis

## Abstract

*Heteroplexis sericophylla* (Asteraceae) is a rare and vulnerable species endemic to China. Here, we report and characterize the complete chloroplast genome sequence of *H. sericophylla* based on Illumina paired-end sequencing data. The complete plastid genome was 152,629 bp in length, which contained two inverted repeats (IRs) of 24,954 bp separated by a large single-copy (LSC) and a small single-copy (SSC) of 84,427 bp and 18,294 bp, respectively. The cpDNA contains 131 genes, comprising 85 protein-coding genes, 37 tRNA genes, 8 rRNA genes and one processed pseudogene. The overall GC content of the plastome is 37.3%. The phylogenetic analysis of 17 selected chloroplast genomes demonstrated that *H. sericophylla* was close to congeneric species *H. incana*.

*Heteroplexis sericophylla* Y. L. Chen, an annual herb which belongs to the tribe of Astereae in Asteraceae, is a rare and vulnerable species endemic to Guangxi Zhuang Autonomous Region of China. Due to *H. sericophylla* only grows on the limestone peak and has an extremely narrow distribution range, its population is very limited (Shi et al. 2018). Hence, *H. sericophylla* is treated as ‘vulnerable’ in China (Fu 1992) and it has been registered on the China Species Red List (Wang and Xie 2004). It is thus urgent to take effective measures to conserve this vulnerable and rare species. Herein, we report and characterize the complete plastome of *H. sericophylla* based on Illumina paired-end sequencing data, which will contribute to the further studies on its genetic research and resource utilization. The annotated cp genome of *H. sericophylla* has been deposited into GenBank with the accession number ML942054.

In this study, *H. sericophylla* was sampled from in Guangxi Zhuang Autonomous Region of China, located at 110°18′05″E, 25°04′47″N. A voucher specimen (Y.-C. Shi et al. H006) was deposited in the Guangxi Key Laboratory of Plant Conservation and Restoration Ecology in Karst Terrain, Guangxi Institute of Botany, Guangxi Zhuang Autonomous Region and Chinese Academy of Sciences, Guilin, China. The experiment procedure is as reported in Zhang et al. (2019). Around 2 Gb clean data were used for the cp genome de novo assembly by the programme NOVOPlasty (Dierckxsens et al. 2017) and direct-viewing in Geneious R11 (Biomatters Ltd., Auckland, New Zealand). Annotation was performed with the program Plann (Huang and Cronk 2015) and Sequin (http://www.ncbi.nlm.nih.gov/).

The chloroplast genome of *H. sericophylla* is a typical quadripartite structure with a length of 152,629 bp, which contained two inverted repeats (IRs) of 24,954 bp separated by a large single-copy (LSC) and a small single-copy (SSC) of 84,427 bp and 18,294 bp, respectively. The cpDNA contains 131 genes, comprising 85 protein-coding genes, 37 tRNA genes, 8 rRNA genes, and 1 processed pseudogene. Among the annotated genes, 15 of them contain one intron (*atp*F, *pet*B, *pet*D, *ndh*A, *ndh*B, *rps*16, *rpoC*1, *rpl*16, *rpl*2, *trn*A-UGC, *trn*I-GAU, *trn*G-UCC, *trn*K-UUU, *trn*L-UAA and *trn*V-UAC), and three genes (*clp*P, *rps*12 and *ycf*3) contain two introns. The overall GC content of the plastome is 37.3%, which is unevenly distributed across the whole chloroplast genome.

To identify the phylogenetic position of *H. sericophylla*, phylogenetic analysis was conducted. A neighbour-joining (NJ) tree with 1000 bootstrap replicates was inferred using MEGA version 7 (Kumar et al. 2016) from alignments created by the MAFFT (Katoh and Standley 2013) using plastid genomes of 17 species. It showed the position of *H. sericophylla* was close to congeneric species *H. incana* (Figure 1). Our findings can be further used for population genomic and phylogenomic studies of *Heteroplexis*. It will also provide fundamental data for the conservation, utilization and management of this vulnerable and rare species.

**Figure 1.**
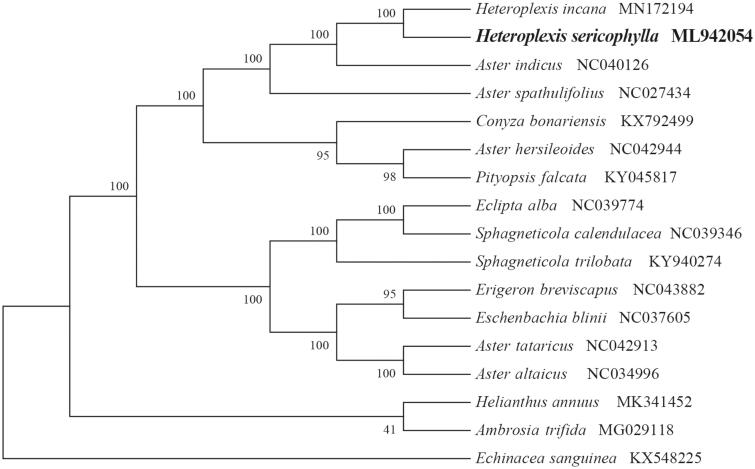
NJ phylogenetic tree of *H. sericophylla* with 16 species was constructed by chloroplast plastome sequences. Numbers on the nodes are bootstrap values from 1000 replicates. *Echinacea sanguinea* was selected as outgroups.
